# Cross-sectional validation of the Aging Perceptions Questionnaire: a multidimensional instrument for assessing self-perceptions of aging

**DOI:** 10.1186/1471-2318-7-9

**Published:** 2007-04-26

**Authors:** Maja Barker, Ann O'Hanlon, Hannah M McGee, Anne Hickey, Ronan M Conroy

**Affiliations:** 1 Department of Psychology, Division of Population Health Sciences, Royal College of Surgeons in Ireland, Dublin, Ireland

## Abstract

**Background:**

Self-perceptions of aging have been implicated as independent predictors of functional disability and mortality in older adults. In spite of this, research on self-perceptions of aging is limited. One reason for this is the absence of adequate measures. Specifically, there is a need to develop a measure that is theoretically-derived, has good psychometric properties, and is multidimensional in nature. The present research seeks to address this need by adopting the Self-Regulation Model as a framework and using it to develop a comprehensive, multi-dimensional instrument for assessing self-perceptions of aging. This study describes the validation of this newly-developed instrument, the Aging Perceptions Questionnaire (APQ).

**Methods:**

Participants were 2,033 randomly selected community-dwelling older (+65 yrs) Irish adults who completed the APQ alongside measures of physical and psychological health. The APQ assesses self-perceptions of aging along eight distinct domains or subscales; seven of these examine views about own aging, these are: timeline chronic, timeline cyclical, consequences positive, consequences negative, control positive, control negative, and emotional representations; the eighth domain is the identity domain and this examines the experience of health-related changes.

**Results:**

Mokken scale analysis showed that the majority of items within the views about aging subscales were strongly scalable. Confirmatory factor analysis also indicated that the model provided a good fit for the data. Overall, subscales had good internal reliabilities. Hierarchical linear regression was conducted to investigate the independent contribution of APQ subscales to physical and psychological health and in doing so determine the construct validity of the APQ. Results showed that self-perceptions of aging were independently related to physical and psychological health. Mediation testing also supported a role for self-perceptions of aging as partial mediators in the relationship between indices of physical functioning and physical and psychological health outcomes.

**Conclusion:**

Findings support the complex and multifaceted nature of the aging experience. The good internal reliability and construct validity of the subscales suggests that the APQ is a promising instrument that can enable a theoretically informed, multidimensional assessment of self-perceptions of aging. The potential role of self-perceptions of aging in facilitating physical and psychological health in later life is also highlighted.

## Background

The relationship between self-perceptions of aging and health-related outcomes has been documented through an array of qualitative and quantitative studies. Self-perceptions categorised as positive or negative have been found to have a differential impact on functional health at 18-year follow-up [1] and even more importantly, on rates of mortality at 23-year follow-up [2]. Research has also shown that beliefs about aging predict cause-specific mortality, i.e. individuals with positive self-perceptions of aging were less likely to die of respiratory causes than individuals with negative self-perceptions of aging [3]. Relationships between self-perceptions of aging and psychological health outcomes such as life-satisfaction [4], quality of life [5], loneliness [6] and depression [7], have also been documented. Furthermore, at a behavioral level, self-perceptions of aging are related to the adoption of health-promoting behaviors [8], and coping strategies [9]. Such findings are particularly important given that negative perceptions and beliefs about aging might be amenable to change [10] and as such could be considered in interventions to facilitate physical and psychological health in later life.

Despite the merit of investigating self-perceptions of aging, the number of reliable and valid quantitative measures is limited [11]. Furthermore, measures with adequate psychometric properties are often either primarily evaluative, i.e. illustrating the extent to which perceptions are positive or negative, or they focus on single dimensions of the aging experience such as anxiety about aging or felt-age. Although such measures shed light on individual dimensions of aging, they do not offer a comprehensive representation of the aging experience. It is now recognised, however, that the experience of aging is complex and multi-faceted, and that there is a need for a multi-dimensional perspective that embraces this complexity [12,5]. In the context of assessment this suggests that there is a need to develop a comprehensive measure that logically coordinates individual dimensions that hereto, have been examined in isolation. Such a measure would facilitate a multi-dimensional and holistic representation of the aging experience.

In tandem with the need to develop a multi-dimensional instrument to facilitate our understanding of the complexity of the experience of aging, is the need to synthesize this information using relevant conceptual frameworks [13]. Developing an instrument that is theory-based would provide a resource that would enable researchers and clinicians to systematically understand, predict and if necessary, modify self-perceptions of aging.

### The self-regulation model and perceptions of aging

Adopting a self-regulation framework for conceptualizing the experience of aging is potentially very useful. Typically, models of self-regulation focus on the self-regulation of behavior or the self-regulation of experience [14] and the majority are either generic in nature [15] or specifically applied to health or illness [16]. One framework that has been widely used to examine the self-regulation of experience, specifically the experience of health and illness is Leventhals' self-regulation model (SRM) [16]. A key assumption of this model is that an individual forms a representation of their health threat or illness that can be divided into a series of logical themes or dimensions: identity (beliefs about the label and nature of the illness and the link with symptoms), timeline (beliefs about the duration and course of the illness, specifically whether it is acute, chronic or cyclical in nature), consequences (beliefs about the impact of the illness on one's life), control (beliefs about personal ways of managing one's illness), cause (beliefs about the likely cause of the illness) and emotional representations (emotional response generated by the illness). These dimensions have been operationalized in a self-report questionnaire, the Illness Perception Questionnaire (IPQ) [17] and it's revised version, the IPQ-R [18]. These measures are widely used in health psychology research and have yielded data which is both informative and predictive [19].

To date, while not inherently a model of health or illness, the SRM has only been applied in the context of health threat. It has been proposed, however, that the same concepts and models can be used to study adaptation to different stressors [20]. The underlying notion is that stressors, no matter, how diverse they may be, can be described in terms of the extent or intensity of the demand they make upon the individuals adaptive resources and/or the individuals coping resources or sense of personal efficacy. While aging is a normal life stage and not an illness or an event that would be included in a traditional list of life stressors, it does however place demands on an individual's resources and entails challenges which need to be managed and adapted to [21]. As such, it is reasonable to propose that the SRM framework may be useful for studying adaptation in the context of aging. Building upon SRM principles and a review of the literature on the experience of aging SRM dimensions have been translated directly into an aging context. These aging dimensions are as follows:

*Identity: *beliefs about ageing in the context of health, specifically the link between aging and health-related changes. Specific health-related changes consider musculoskeletal, cardiovascular, pulmonary, neurological, psychiatric, and general domains of functioning [22] (e.g. 'loss of balance'). Older adults may associate ageing with health-related changes and more specifically with physical decline. Furthermore, it has been proposed that the tendency to attribute health-related changes or physical decline to aging may be inappropriate and may have consequences in that it directs the attention of the older person away from real disease and/or environmental factors affecting health [23,24]. Such a proposal warrants investigation.

*Timeline: *issues relating to an individual's awareness of aging and their experience of the process over time. This has two sub-dimensions: timeline chronic (the extent to which awareness of one's age or aging is chronic in nature, e.g. 'I always classify myself as old'), and timeline cyclical (the extent to which one experiences variation in awareness of aging, e.g. 'I go through phases of feeling old'). Chronic awareness of one's own aging is related to the concept of 'age identification' [25] and this has been associated with inactivity [25] and poor health [27]. The concept of cyclicality has received little empirical attention to date.

*Consequences: *beliefs about the impact of aging on one's life across a variety of domains. This has two sub-dimensions: consequences positive (e.g. 'As I get older I get wiser') and consequences negative (e.g. 'Getting older makes everything a lot harder for me'). The assumption of positive aging consequences has been linked with greater creativity [28] and greater subjective well-being [5]. Conversely, the assumption of negative aging consequences has been linked with depression [7] and lower subjective well-being [5].

*Control: *beliefs about personal ways of managing one's experience of aging. This has two sub-dimensions: control over positive experiences (e.g. 'The quality of my social life in later years depends on me') and control over negative experiences (e.g. 'Immobility in later life is not up to me') relating to aging. The significance of control gains support from research demonstrating the importance of primary and secondary control strategies in later life [29] with the former referring to peoples' efforts to control their environment to suit their needs, and the latter referring to the various ways in which people reinterpret themselves or their situation so as not to become overwhelmed when the environment will not yield to their influences. Further support for control is derived from research which shows that having a strong sense of control over one's development can facilitate well-being throughout the life cycle [30]. Although control in the context of later life has received attention, to date, beliefs about control over positive and negative experiences of aging have not been empirically investigated.

*Emotional representations: *the emotional response generated by aging. It is specifically represented by negative emotions such as worry, anxiety, depression, fear, anger, and sadness (e.g., 'I get depressed when I think about getting older'). Negative emotional responses to aging have been associated with negative changes in physical and functional health [31] and with maladaptive coping [32].

### Development of the Aging Perceptions Questionnaire (APQ)

These dimensions were used to develop a multi-dimensional assessment tool. In developing this instrument, focus groups were conducted with older adults to consider the relevance of the theoretical framework for understanding self-perceptions of aging and to develop items for the proposed Aging Perceptions Questionnaire (APQ). Participants were asked about their experiences of aging with specific questions focusing on perceptions of aging using dimensions from the SRM as it had been adapted for an aging context. Interviews were transcribed and thematically analysed. Findings were consistent with the newly translated aging dimensions, i.e., the SRM was a useful framework for gaining insight into self-perceptions of aging. From this data a pool of items was developed and reviewed by a team of 16 expert clinicians and researchers in aging. Judgments about whether to include an item were based on several factors: the degree to which a given item reflected how a particular aging perception was defined (content validity); the logic of a given item, specifically its meaning and readability for participants; and the validity and content coverage of items within the broader aging literature. A preliminary study was conducted to test the psychometric properties of this instrument with a sample of community-dwelling people aged 65 and over (n = 129). Psychometric, conceptual and practical criteria were used to identify items to be retained. The structural and psychometric properties of the subscales were subsequently investigated and found to have good structural and psychometric properties. Overall, the findings indicated that the APQ was a promising instrument for assessing self-perceptions of aging (further information on this pilot study can be obtained from the corresponding author).

A second preliminary study was also conducted to investigate the test-retest reliability and convergent validity of the APQ. Participants were community-dwelling adults aged 65 and over (n = 143). Test-retest time ranged from 7 to 42 days (Mean = 13 days, SD 7.3). Intra-class correlation coefficients were 0.78 (timeline chronic), 0.79 (timeline cyclical), 0.70 (consequences positive), 0.83 (consequences negative), 0.83 (emotional representations), 0.76 (control positive), 0.61 (control negative), and 0.75 (identity). The average test-retest reliability of the APQ was 0.76, considered to be an index of excellent test-retest reliability (Rosner, 1995). Correlations between the APQ and uni-dimensional measures of the aging experience also supported both the convergent and divergent validity of individual APQ subscales. For instance, timeline chronic and timeline cyclical subscales were significantly correlated with 'felt age' indicating that a chronic awareness of aging and variation in relation to one's experience of aging was related to the feeling that one is older than one's chronological age (r = -.35, p < .001; r = -.35, p < .001 respectively). Higher identity scores were also significantly associated with lower scores on the expectations regarding physical health domain of the Expectations Regarding Aging Survey (ERA-12) [31] (r = -.23, p < .05). The emotional representations subscale was significantly correlated with the 'psychological concerns' and 'fear of losses' (r = .32, p < .001; r = .40, p < .001, respectively) subscales of the Anxiety about Aging Scale [34] but not with the 'fear of old people' and 'physical appearance' subscales. APQ control dimensions were correlated with scores on the personal control over development scale [35] with results showing that control positive was significantly associated with personal control over development (r = .29, p < .01), while control negative was not. The consequences negative subscales were correlated with the Attitude towards own aging scale of the PGCMS [36,37] (r = -.20, p < .05). Consequences positive and consequences negative were both significantly associated with scores on the Expectations Regarding Aging Survey (ERA-12); high levels of perceived negative aging consequences were significantly associated with more negative attitudes towards aging and with more negative expectations on all ERA domains (range from -.25 to -.33, p < .001 for all correlations), consequences positive, however, was not related to expectations regarding aging.

In conclusion, the investigation of test-retest and convergent validity showed modest associations between the APQ and measures of individual aging-related dimensions. Testing the convergent validity of the APQ was challenging, namely, because it was not specifically designed to replicate the existing methods against which it was being compared. The largest correlation coefficient between any APQ dimension and an established measure was .40 which suggests that none of the established measures perfectly capture the constructs underlying the APQ. This finding was thought-provoking as it further highlighted the complexity of the aging experience and the wide array of meaningful constructs that need to be considered in any research or clinical paradigm.

### Present research

The present study aimed to evaluate the Aging Perceptions Questionnaire (APQ) with a national, randomly selected, representative sample of community dwelling older adults. Specific objective were to provide further support for APQ psychometric properties and further substantiate the construct validity of APQ subscales, specifically by testing their associations with indices of physical and psychological health.

## Methods

### Sample

2,033 people (age range 65–102 years) participated in the present study. This study had been approved by the institution's research ethics committee. Participants were identified by the Register of Electors or postal address files in the Republic of Ireland and Northern Ireland respectively. This enabled random selection of private households. Eligibility was for those aged 65 years or more, living in a private household. Survey fieldwork was carried out by professionally trained survey research staff who conducted interviews in participants' own homes. Potential participants were given written information about the study, and those who agreed to partake signed a consent form. Survey quality was controlled by means of briefing sessions, interviewer training and monitoring, and post-survey checking.

Completion rate for interviews was 68%. More detailed information on the sample and its similarity to census data in Ireland can be seen elsewhere [38,39]. For descriptive participant information see Table 1. Data was collected as part of a multidisciplinary Healthy Aging Research Programme (HARP) which aims to examine the experience of health and aging among community-dwelling older adults in Ireland.

### Measures

#### Socio-demographic variables

Age, gender, education, marital status and household income were recorded.

#### Self-perceptions of aging

The Aging Perceptions Questionnaire (APQ) (see Additional file 1) has eight subscales. At the time of validation thirty-five items comprised the seven sub-scales which look at views about aging. Following validation (to be described) three items were removed. The final key sub-scales are timeline chronic (Items 1–5), timeline cyclical (Items 27, 28, 30, 31, 32), consequences positive (Items 6, 7, 8), consequences negative (Items 16–20), control positive (Items 10, 11, 12, 14, 15), control negative (Items 21–24), and emotional representations (Items 9, 13, 25, 26, 29). An identity subscale is also included (Id1-Id17). For the seven key subscales items are rated on a 5-point scale ranging from strongly disagree to strongly agree. With the exception of control negative, subscales are scored from 1 to 5. The mean score for each subscale is calculated. Higher scores are indicative of greater endorsement of a specific perception.

The identity subscale examines the experience of health-related changes and consists of 17 possible health-related changes. Participants are firstly asked to indicate whether they have experienced these changes over the last 10 years (1 = Yes, 0 = No). When the response is affirmative participants are then asked whether they attribute these changes to getting older (1 = Yes, 0 = No). Scores on these subscales can range from 0–17. The percentage of health-related changes attributed to aging is then tabulated as a proportion of the number of health-related changes experienced. This yields an identity score. Scores for identity can range from 0 to 100 (for information on scoring all sub-scales see Additional file 2). The APQ has a Flesch reading ease of 61.8 and a Flesch-Kincaid reading level of 8.3 which suggests good instrument readability.

#### Physical health status

Level of functional disability and number of comorbidities were used as indices of physical health status. Functional disability was assessed using the Health Assessment Questionnaire Disability Index (HAQ-DI) [40]. Scores range from zero (self-sufficient) to three (severely disabled). Studies corroborate the validity and reliability of the HAQ-DI [41]. A co-morbidity index (CMI) was also calculated by coding self-reported medical problems/illness in accordance with different organs or systems. Coding cross-checks were performed by two researchers and a research nurse. Inter-rater reliability was 100%. Organs or systems were those that have been utilized in the modified cumulative illness rating scale (CIRS) [42]. The CMI index represents the number of medical problems identified by the participant. Higher CMI scores have been shown to reflect greater medical complexity [43].

#### Psychological health

Depressive symptoms were used as an index of psychological health and were assessed using the seven-item depression subscale of the Hospital Anxiety and Depression Scales (HADS-D) [44]. The HADS-D is widely used [45] and performs well in assessing severity and caseness of depression in clinical and general populations [46]. Higher scores on the depression sub-scale of the HADS-D reflect higher levels of depression.

### Statistical analysis

Mokken scale analysis for polytomous items (msp) was conducted for the seven key subscales to determine whether items were scalable, i.e., items within scales were assessing the same underlying trait or APQ dimension. Mokken scaling is a nonparametric, iterative scale-building technique which attempts to build the smallest set of internally-consistent scales from a given item pool. The identity scale was analysed separately as it differs in orientation from the seven key subscales looking at views about aging. Specifically, the identity scale examines the experience of health in the context of aging while the seven key subscales examine views about aging in a variety of contexts, e.g. physical, and social. Confirmatory factor analysis was then conducted with the views about aging subscales to examine the relative fit of the seven-factor solution to the data.

Reliability for each of the APQ subscales was measured using Cronbach's alpha. The construct validity of the APQ was also assessed. First, the validity of the identity subscale was tested by using the Wilcoxon signed ranks test to determine whether there were differences between the health-related changes experienced and the health-related changes attributed to aging, and also by investigating the frequencies with which different health-related changes were attributed to aging. Pearson correlations were conducted to examine the relationship between all APQ subscales to determine the logical consistency of these relationships. Following this hierarchical regression analyses and mediation testing were conducted to examine the independent contribution of APQ variables to physical and psychological health, namely functional disability (HAQ-DI), and depression (HADS-D). Analyses were conducted with SPSS for Windows (Version 12.0), EQS structural equation modelling, and Mokken scale analysis for polytomous items using a procedure written by Jean-Benoit Hardouin for Stata Release 9 [47,48].

## Results

Results on the psychometric properties of the APQ, namely item and scale evaluation are presented first, followed by results relating to construct validity.

### Evaluation of items and subscales in the APQ

Using a Loevinger coefficient of homogeneity (H) of .30 as a critical level for scalability in Mokken scale analysis, all items for timeline chronic (H = .62), timeline cyclical (H = .65), consequences negative (H = .56), control positive (H = .48), and emotional representations (H = .55) were seen to be strongly scalable. For control negative, one item failed to reach the criterion for scalability and was excluded. The other 4 items were strongly scalable (H = .46). For consequences positive two items were excluded, the remaining three items were strongly scalable (H = .45). Thus 32 items comprised the final 'views about own aging' section of the APQ. Confirmatory factor analysis was then conducted to examine the relative fit of the seven-factor solution to the data. The resulting model was found to have a good fit (χ2 = 2788, DF = 496, CFI = .91, IFI = .91, GFI = .91, RMSEA = .05, p < .001) indicating that each subscale addresses a unique dimension of an individual's experience of aging. (Other possible explanations for the data were examined but found not to provide a good fit, e.g. a one factor model, and multiple two factor models.)

### Subscale distributions and internal reliability

The distribution and reliability of APQ subscales can be seen in Table 2. With the exception of the consequences positive subscale Cronbach alpha coefficients for subscales were above 0.70 and typically exceeded 0.80. Internal reliability was not tabulated for the Identity subscale as this scale consists of a series of disparate health-related changes. All of the subscales had less than 5% missing data. None of the APQ 'views about aging' subscales had notable floor or ceiling effects. The identity scale did however demonstrate a ceiling effect: 37.7% of participants who had experienced health-related changes attributed all of these health-related changes to aging.

### Construct validity

The Wilcoxon signed ranks test provided support for the conceptual difference between the sum of health-related changes experienced and the sum of health-related changes attributed to aging (z = 30.402, p < .0001, two-tailed). The frequencies with which different health-related changes were experienced, and the frequencies with which *experienced *health-related changes were attributed to aging i.e. identity, were also tabulated (see Table 3). All 17 health-related changes had been experienced by a percentage of participants thus supporting the validity of the range of items in this subscale. The most frequently experienced health-related changes were slowing down (71.9%), vision changes (45.4%) and loss of strength (44.5%) The least frequently reported health-related change was depression (14.0%). All of the experienced health-related changes were attributed to aging by some participants. The change most frequently attributed to aging was 'slowing down'; 65.4% of participants who believed that they had slowed down in the last ten years attributed this to aging, while the least frequently attributed change was 'depression; 7.7% of participants who had experienced depression in the last ten years attributed this specifically to aging.

Significant relations were seen between APQ components (see Table 4). For instance, control negative was negatively associated with a chronic (r = -.41, p < .001) as well as a cyclical timeline (r = -.32, p < .001) indicating that individuals who assumed greater control over negative experiences and outcomes relating to aging were less aware of aging and experienced less variation in their experience of the process. In keeping with this, control positive was also positively associated with positive aging consequences (r = .28, p < .001). Thus individuals who felt they had control over negative experiences relating to aging were also more likely to see it as having positive consequences.

Data on physical and psychological health (functional disability and depression) also provided the opportunity to test the construct validity of APQ subscales. Multiple linear regression models were developed with depression and functional disability in order to examine the independent contributions of measures when entered together. Socio-demographic variables were entered on the first step, indices of health status were entered on the second step and perceptions of aging were entered on the third step. This sequence was adopted in order to determine the contribution of APQ variables even after other more traditional measures had been accounted for. Only those variables that were significantly associated with depression and functional disability as a result of preliminary bivariate analyses were considered. Table 5 displays the standardised regression coefficients (β), R, R^2 ^and adjusted R^2 ^after entering all variables. R was significantly different from zero at the end of each entry.

In the context of depression, the first model (socio-demographic variables) explained 7.1% of the variance in depression scores (F _(4,1950_) = 38.464, p < .0001). Significant variables were age and education; being older, and having a lower level of education were associated with higher depression scores. Model 11 (health status) explained an additional 22.2% of the variance in depression scores (F _(6,1948) _= 136.021, p < .0001). Higher levels of functional disability and a greater number of comorbidities were associated with higher levels of depression. With the addition of health indices, education was no longer significant. Model 111 (self-perceptions of aging) explained an additional 14.9% of the variance (F _(14,1940) _= 111.338, p < .0001) in depression scores. Significant APQ variables were timeline chronic, emotional representations, control positive, consequences positive, consequences negative, and identity. A more chronic awareness of aging, a more negative emotional response to aging, more perceived negative aging consequences, and attributing experienced health-related changes to aging were associated with higher depression scores. Conversely, beliefs about control over positive experiences relating to aging and the assumption of positive aging consequences were associated with lower depression scores. The largest significant beta coefficient for APQ variables was seen for control positive. With the introduction of APQ variables the co-morbidity index was no longer significant in explaining variance in depression. Furthermore, the beta coefficient for functional disability remained significant but decreased by 30% (from 0.465 to 0.324). This suggests that self-perceptions of aging may play a mediating role in the relationship between health status and depression.

To consider this further another regression was conducted, this time with the omission of indices of health status. In this analysis the total variance explained was 36.5% (Adjusted R^2^), thus a decrease of only 7.6% (from 44.1% to 36.5%). Model 1 (socio-demographic variables) explained 7.2% of the variance (F _(3,1956) _= 51.393, p < .0001) and Model 11 (self-perceptions of aging) explained 29.3% of the total variance in depression scores (F _(11,1956) _= 103.120, p < .0001). This is also supportive of a mediatory role for APQ dimensions.

Formal mediation testing was subsequently conducted to determine whether individual APQ dimensions mediate the relationship between indices of physical health and depression. The mediating role of individual APQ dimensions in the relationship between co-morbidity (independent variable) and depression (dependent variable) and between functional disability (independent variable) and depression (dependent variable) was examined by investigating correlation coefficients and changes in beta-coefficients when entering individual APQ variables in a series of regression models. Only those APQ dimensions that had emerged as significant when they had been entered simultaneously were considered. These were timeline chronic, emotional representations, control positive, consequences positive, consequences negative, and identity.

In the relationship between functional disability and depression, the first three conditions for mediation specified by Baron and Kenny [49] were met by APQ dimensions. Each APQ variable was subsequently tested to see if it fulfilled the fourth condition for mediation; the effect of including each APQ variable in hierarchical linear regressions where level of functional disability was the independent variable and depression was the dependent variable was assessed. Sobel's tests supported a mediating role for individual APQ variables (timeline chronic, z = 11.09, p < .00001; emotional representations, z = 10.55, p < .00001; control positive, z = 10.93, p < .00001; consequences negative, z = 11.63, p < .00001; consequences positive, z = 2.99, p < .001; Identity, z = 3.51, p < .01). As can be seen from Sobel's tests the largest mediating role was assumed by consequences negative, followed by timeline chronic, control positive, emotional representations, and consequences positive.

In investigating the relationship between co-morbidity and depression, the first three conditions for mediation specified by Baron and Kenny were satisfied.

Subsequent testing for the fourth condition of mediation showed that the inclusion of APQ dimensions yielded significant reductions in the beta-coefficients for co-morbidity with Sobel's tests supporting a mediating role for individual APQ variables (timeline chronic, z = 9.53, p < .00001; emotional representations, z = 9.91, p < .00001; control positive, z = 8.72, p < .00001; consequences negative, z = 11.35, p < .00001). In all mediation analyses although beta coefficients for co-morbidity were significantly reduced, they did remain significant which once again suggests partial as opposed to full mediation. Sobel's tests indicated that the largest mediating role was assumed by consequences negative, followed by emotional representations, timeline chronic, and control positive.

Hierarchical regression analysis was also conducted with functional disability (HAQ-DI) scores as an outcome measure. Table 6 displays the standardised regression coefficients (β), R, R^2 ^and adjusted R^2 ^after entering all variables. R was significantly different from zero at the end of each entry. After the first entry with socio-demographic variables in the equation, 13.3% of the variance in functional disability was accounted for (F _(4,1958) _= 76.496, p < .0001. Significant variables were sex, age, and education; being female, being older, and having a lower level of education were associated with greater functional disability (HAQ-DI). An index of co-morbidity was added in the second model and this explained an additional 14.0% of the variance (F _(5,1957) _= 148.618, p < .0001). Co-morbidity was significantly positively associated with HAQ-DI scores i.e. more co-morbidities were associated with more severe disability. The third model introduced self-perceptions of aging into the equation: This set of indicators explained an additional 7.4% of the variance in functional disability (F _(13,1949) _= 81.330, p < .0001). Significant variables were timeline chronic, emotional representations, control positive and consequences negative. A more chronic experience of aging, a more negative emotional response to aging and more perceived negative aging consequences were associated with higher disability levels. Conversely, perceptions of control over positive experiences relating to aging were associated with less functional disability. The largest significant beta coefficient for APQ variables was seen for consequences negative. Socio-demographic variables remained significant. The co-morbidity index continued to be a significant explanatory variable however its contribution was reduced by 27%. This suggests that APQ variables may partially mediate the relationship between co-morbidity and functional disability (HAQ-DI).

In order to investigate this further, another regression was conducted with the co-morbidity index omitted. In this analysis the total variance explained was 28.2%, thus a decrease of only 6.5% (from 34.7% to 28.2%). In this analysis socio-demographic variables accounted for 13.3% of the variance in functional disability (F _(4,1958) _= 76.496, p < .0001) while self-perceptions of aging explained an additional 14.9% of the variance (F _(12,1950) _= 65.140, p < .0001). This supports the proposal that self-perceptions of aging may play a mediating role in the relationship between co-morbidity and functional disability.

In investigating the relationship between co-morbidity and functional disability, the first three conditions for mediation were satisfied. Subsequent testing for the fourth condition of mediation showed that the inclusion of APQ dimensions yielded significant reductions in the beta-coefficients for co-morbidity with Sobel's tests supporting a mediating role for individual APQ variables (timeline chronic, z = 8.40, p < .00001; emotional representations, z = 7.88, p < .00001; control positive, z = 7.86, p < .00001; consequences negative, z = 10.20, p < .00001). In all mediation analyses although beta coefficients for co-morbidity were significantly reduced, they did remain significant which once again suggests partial as opposed to full mediation. Sobel's tests indicated that the largest mediating role was assumed by consequences negative, followed by timeline chronic, emotional representations, and control positive.

## Discussion

This study describes the development of a theoretically-informed, multidimensional instrument to assess adult perceptions of their own aging. Testing and validation has been with a large representative sample of older Irish adults. Results support the multi-dimensional nature of the experience of aging and of the dimensions outlined in the SRM applied in an aging context. The reliability and validity of the APQ has also been demonstrated by the generally good internal consistencies of subscales, by logically consistent subscale interrelations and by relations between APQ subscales and physical and psychological health indices. Findings also highlight the theoretical importance of specific perceptions and of their potential role in physical and psychological health. The use of a theoretical framework has been valuable for determining how individual dimensions relate to each other and what their comparative role is in physical and psychological health.

Results relating to timeline subscales of the APQ showed that there were interrelations between these and other APQ subscales. Timeline chronic was positively associated with timeline cyclical which suggest that individuals who were chronically aware of their aging also experienced more variation in their experience of the process. Both chronic and cyclical timelines were associated with more negative emotional responses to aging, lower levels of perceived control over experiences relating to aging, more negative aging consequences, and higher aging identity scores. A cyclical timeline was also associated with fewer positive aging consequences. The logical consistency of these interrelations is supported by findings highlighting the independent role that a chronic timeline might play in physical and psychological health. These findings corroborate previous research showing how a chronic awareness of aging might be maladaptive [26,27].

Results relating to aging consequences also highlighted the potential importance of these beliefs. APQ interrelations showed that individuals who identified a high degree of negative aging consequences were also more likely to endorse potentially maladaptive perceptions such as decreased perceptions of control and a more negative emotional response to aging. Conversely, individuals who identified a high degree of positive aging consequences were more likely to endorse other potentially adaptive perceptions such as perceived control over positive and negative aging experiences. The potential significance of consequences dimensions is also derived from their relationship with physical and psychological health; consequences negative accounted for variation in functional disability and depression after traditional explanatory variables had been controlled for. Comparatively, there was variation in the role played by consequences positive, specifically in that it explained variation in depression but not in functional disability. These findings corroborate previous research and provide support for the construct validity of APQ dimensions [1,2] while at the same time highlighting the need for looking in detail at constructs such as consequences.

Considering APQ control dimensions results showed that control positive and control negative were inversely associated with emotional representations, timeline, chronic, timeline cyclical, consequences negative, and identity. Both control dimensions were also positively associated with the endorsement of positive aging consequences. Support for the adaptive value of control [30] is further highlighted through associations between perceived control and indices of physical and psychological health. There was variation, however, in the role played by these dimensions; control positive but not control negative accounted for variation in functional disability and depression. This suggests that encouraging a sense of control over positive as opposed to negative aging experiences might be important for facilitating better personal health in later life. Overall, the present findings corroborate suggestions that perceived control may influence adaptive outcomes in later life [50] but they also elucidate the specific control dimension that might exert an effect.

Results relating to the emotional representations subscale of the APQ showed that individuals who had strong negative emotional responses to aging also endorsed negative perceptions such as a chronic timeline. Conversely, individuals with less negative emotional responses to aging also endorsed positive perceptions such as control and positive aging consequences. The potential maladaptiveness of emotional representations was highlighted by the significant role that this dimension played in functional disability and depression. This is consistent with previous research showing associations between negative emotional responses to aging and late life maladaptive outcomes [32,31].

Finally, results relating to the identity subscale demonstrated that individuals who ascribed experienced health-related changes to aging also endorsed other more negative perceptions such as a more chronic awareness of aging, more variation in the experience of the aging process (timeline cyclical), a negative emotional response, more perceived negative aging consequences, and lower levels of control over experiences relating to aging. Identity scores were not, however, significant in explaining functional disability or depression.

One limitation of this study is its cross-sectional design. This means that inferences about associations between self-perceptions of aging and health outcomes and their potential mediating roles cannot be confirmed. Replication with a prospective design is needed to determine the nature of the relationships. Moreover, without any external validating data, convergent validity, i.e. the convergence of related scales and instruments, cannot be determined. As such, although the APQ is measuring distinct constructs, without further evaluation it is not possible to ensure that the scale is measuring what it purports to do. Nevertheless, findings suggest that the APQ has great potential as an instrument for assessing self-perceptions of aging. They also highlight the value of SRM dimensions. Endorsing the SRM as a conceptual framework means that the APQ can address limitations pertaining to existing measures, by offering the multidimensionality and specificity necessary to gain detailed, comprehensive assessments of the subjective experience of aging. In practical terms, the APQ is also useful as it is concise and does not require specialized personnel (i.e., psychologists, geriatricians or social workers) to administer it.

One potential concern about the use of the SRM is its prior association with the assessment of illness and thus conceptually of negative outcomes or decline. In adapting the SRM, we did not however assume that aging was a process of decline. We do acknowledge that within the context of illness, the model appears as a model of decline. This, however, is more a reflection of the items that are used in the Illness Perception Questionnaire (IPQ), and the fact that most participants associated only negative consequences with illness, rather than a reflection of the actual assumptions that the model itself endorses. In the context of aging, the model was not assumed to be a model of decline although a number subscales reflect decline because our older adult participants automatically generated negative items. To prevent the questionnaire from being solely based on decline, we specifically attempted to introduce a positive stance whereby we challenged people to think about positive aspects of aging, e.g. positive consequences.

Another reason why we consider the SRM to be a useful framework relates to the emphasis that it places on understanding experience from the perspective of the individual. This perspective is consistent with traditional, cognitive models of the behavioral process that represent the individual as a common-sense 'scientist' trying to make sense of his/her world. At the core of this cognitive approach is the view that individuals construct models, internal representations, or schema, which reflect their pooled understanding of previous experiences and are used for interpreting new ones and planning their behavior. This approach is also consistent with an interpretive approach to aging and the life cycle in that it tries to illuminate what aging experiences mean on an individual level. In doing so it reflects the view that an adequate understanding of the aging process can only be gained by accounting for the ways that individuals interpret the meaning of aging.

The APQ can serve in identifying adaptive and maladaptive beliefs regarding aging at individual and group levels. Significant interrelations between APQ dimensions suggest that different dimensions might be targeted in parallel to encourage both a more positive and a less negative perception of aging. Theoretically, perceptions of aging may influence health-related outcomes. The present findings support previous research highlighting the role of self-perceptions of aging in functional health [1] and depression [7]. Importantly, these findings extend previous work by identifying the comparative role played by different self-perceptions of aging. Practically, these findings support the recommendation that beliefs about aging be formulated in cognitive-behavior therapy for late life depression [52]. It is essentially difficult to deny that interventions to facilitate physical and psychological health would be enhanced if they were founded on a thorough systematic understanding of the way in which psychological factors relate to adaptive and maladaptive outcomes. In this regard, the benefit of considering self-perceptions of aging as psychological risk factors and resources is highlighted.

The mechanisms of the potential effects of self-perceptions of aging on disability and depression also need to be considered in further research. In the context of health and illness it has been suggested that illness representations guide individual coping responses [52]. Similarly, it is plausible that self-perceptions of aging might exert an effect on outcomes via a behavioral pathway.

## Conclusion

The APQ is a novel instrument with good psychometric properties to assess self-perceptions of aging. The APQ can contribute to our understanding of the subjective experience of aging and may help in identifying individuals who are at risk because of maladaptive perceptions regarding the aging process. Such information could potentially be used to devise interventions that facilitate a more positive aging experience and more optimal outcomes in later life. The APQ is a simple, flexible method for assessing self-perceptions of aging and it is hoped that it will useful in facilitating theoretical and practical advances in the link between self-perceptions of aging and health-related outcomes.

## Competing interests

The author(s) declare that they have no competing interests.

## Authors' contributions

MB generated conceptual ideas, participated in the design of the study and data collection, performed statistical analyses, interpreted findings, and drafted the manuscript. AOH participated in the refinement of conceptual ideas, participated in the design of the study, participated in statistical analyses, and reviewed the manuscript. HMG participated in the refinement of conceptual ideas, participated in the design of the study, and reviewed the manuscript. AH participated in the refinement of conceptual ideas, participated in the design of the study, and reviewed the manuscript. RC participated in statistical analyses, provided statistical advice, and reviewed the manuscript.

## Pre-publication history

The pre-publication history for this paper can be accessed here:



## Supplementary Material

Additional file 1 Aging Perceptions Questionnaire (APQ). A novel instrument designed to assess individuals' perceptions of their own aging. Click here for file

Additional file 2 Scoring information. Protocol for tabulating APQ subscale scores. Click here for file

## Figures and Tables

**Table 1:** Sample Characteristics (n = 2,033)

**Characteristic**	
Mean Age (years) (SD)	74.1 (6.8)
Gender (%)	
Men	43
Marital status (%)	
Married	44
Widowed	40
Never married/single	12
Separated/divorced	4
Education (%)	
Primary school	60
Secondary school	33
Third-level (diploma/degree)	7
Weekly household Income (€)(M ± SD)	335.70 (190.10)
Functional Disability (HAQ-DI) (M ± SD)	0.50 (0.77)
Depression (HADS-D) (M ± SD)	5.54 (2.85)
Comorbidity Index (CMI)	0.79 (0.95)

**Table 2:** Distributions and Internal Reliability for Subscales of the Aging Perceptions Questionnaire (APQ) (n = 2,033)

	**Item N**	**Range**	**Mean (SD)**	**Median**	**% scoring minimum**	**% scoring maximum**	**Alpha**
***Views about aging***							
Timeline (chronic)	5	4	2.9 (.87)	2.80	1.8	0.8	0.86
Timeline (cyclical)	5	4	2.7(.82)	2.60	2.4	0.2	0.89
Consequences positive	3	4	3.7 (0.59)	4.00	0.2	2.6	0.64
Consequences negative	5	4	3.4 (.74)	3.60	0.2	1.8	0.80
Emotional representations	5	4	2.4 (.74)	2.00	2.8	0.1	0.74
Control positive	5	3.60	3.80 (.54)	4.0	0.2	2.2	0.80
Control negative	4	4.00	2.65 (0.71)	2.50	1.6	0.2	0.73
***Experience of health-related changes ***							
Number of changes experienced	17	17	5.44 (3.68)	5.00	6.2	0.1	-
Number of changes attributed to aging	17	17	3.77 (3.30)	3.00	15.8	0.1	-
Identity: Percentage of experienced changes attributed to aging	-	100	65.0 (37.0)	78.0	15.8	37.7	-

**Table 3:** Participant Experience of Health-Related Changes and Attribution of Experienced Changes to Aging (n = 2,033)

**Health-related change**	**Frequency (%) **	
	**Have experienced health-related change**	**Attributed experienced change to aging **
1) Weight problems	28.2	13.1
2) Sleep problems	35.7	19.8
3) Back problems or slipped disc	29.5	14.2
4) Painful joints	55.4	40.8
5) Not being mobile	27.0	19.5
6) Loss of balance	20.4	12.8
7) Loss of strength	44.5	39.3
8) Slowing down	71.0	65.4
9) Cramps	25.0	17.6
10) Bone or joint conditions	42.8	31.6
11) Cardiac problems	24.8	13.1
12) Ear or hearing problems	25.6	18.8
13) Vision and eyesight changes	44.4	37.4
14) Respiratory problems	20.3	12.2
15) Foot problems	19.3	12.2
16) Depression	13.9	6.6
17) Anxiety	16.2	7.7

**Table 4:** Correlations between APQ Dimensions (n = 2,033)

**APQ Dimensions**	**1**	**2**	**3**	**4**	**5**	**6**	**7**
1. Identity	-	-	-	-	-	-	-
2. Timeline (chronic)	.14**	-	-	-	-	-	-
3. Timeline (cyclical)	.15**	.50**	-	-	-	-	-
4. Consequences positive	.01	-.07**	-.08**	-	-	-	-
5. Consequences negative	.26**	.55**	.42**	-.09**	-	-	-
6. Control positive	-.10**	-.34**	-.24**	.28**	-.32**	-	-
7. Control negative	-.15**	-.41**	-.32**	.06*	-.53**	.26**	-
8. Emotional representations	.12**	.53**	.65**	-.14**	.44**	-.37**	-.38**

** = p < .001, * = p < .01

**Table 5:** Summary of Hierarchical Regression Analysis Explaining HADS-D Depression Scores (n = 2033)

Parameter	Standardised regression coefficients			R	R^2^	Adjusted R^2^
*(I) Socio-demographic variables:*	Model I	Model II	Model III	*.270*	*.073*	*.071*
Age	.233**	.095**	.026**			
Gender	-.002	-.044	-.036			
Education	-.085**	-.019	.007			
Income	-.028	-.014	-.014			
*(II) Physical health indices:*				*.543*	*.295*	*.293*
HAQ-DI		.465**	.324**			
Co-morbidity Index		.082**	.014			
*(III) Self-perceptions of aging:*				*.667*	*.446*	*.442*
Timeline (acute/chronic)			.106**			
Timeline (cyclical)			-.010			
Emotional representations			.144**			
Control positive			-.163**			
Control negative			.004			
Consequences positive			-.067**			
Consequences negative			.117**			
Identity			.105**			

** = p < .001, * = p < .01

**Table 6:** Summary of Hierarchical Regression Analysis Explaining HAQ-DI Disability Scores (n = 2033)

Parameter	Standardised regression coefficients			R	R^2^	Adjusted R^2^
*(I) Socio-demographic indices:*	Model I	Model II	Model III	*.368*	*.135*	*.133*
Gender	.085**	.074**	.071**			
Age	.296**	.294**	.228**			
Education	-.123**	-.085**	-.053**			
Income	-.027	-.017	-.015			
*(II) Physical health indices:*				*.525*	*.275*	*.273*
Co-morbidity		.377**	.276**			
*(III) Self-perceptions of aging:*				*.593*	*.352*	*.347*
Timeline (chronic)			.060*			
Timeline (cyclical)			-.026			
Emotional representations			.087**			
Control positive			-.131**			
Control negative			-.020			
Consequences positive			.001			
Consequences negative			.142**			
Identity			-.033			

** = p < .001, * = p < .01
