# Supplementary material for: Cross-sectional validation of the Aging Perceptions Questionnaire: a multidimensional instrument for assessing self-perceptions of aging
Source: BMC Geriatr. 2007 Apr 26;7:9. doi: 10.1186/1471-2318-7-9 (PMC1868732; doi:10.1186/1471-2318-7-9)
Supplement: Additional file 1 — Aging Perceptions Questionnaire (APQ). A novel instrument designed to assess individuals' perceptions of their own aging. [file 1471-2318-7-9-S1.doc]

***Aging Perceptions Questionnaire (APQ)***

These questions assess your views and experiences of getting older. Since everyone is getting older, these questions can be answered by anyone of any age. There are no right or wrong answers – just your experiences and views. Even if the statement relates to something you do not often think about in relation to yourself, please try to give an indication of your views by answering every question.

| 1. **VIEWS ABOUT AGING** | | | | | |
| --- | --- | --- | --- | --- | --- |
| We are interested in your own personal views and experiences about getting older. Please indicate your views on the following statements (strongly disagree, disagree, neither agree nor disagree, agree, or strongly agree). Circle the response that best describes your view for each statement. | | | | | |
|  | Strongly Disagree | Disagree | Neither Agree Nor Disagree | Agree | Strongly Agree |
| 1. I am conscious of getting older all of the time | _1_ | _2_ | _3_ | _4_ | _5_ |
| 1. I am always aware of my age | _1_ | _2_ | _3_ | _4_ | _5_ |
| 1. I always classify myself as old | _1_ | _2_ | _3_ | _4_ | _5_ |
| 1. I am always aware of the fact that I am getting older | _1_ | _2_ | _3_ | _4_ | _5_ |
| 1. I feel my age in everything that I do | _1_ | _2_ | _3_ | _4_ | _5_ |
| 1. As I get older I get wiser | _1_ | _2_ | _3_ | _4_ | _5_ |
| 1. As I get older I continue to grow as a person | _1_ | _2_ | _3_ | _4_ | _5_ |
| 1. As I get older I appreciate things more | _1_ | _2_ | _3_ | _4_ | _5_ |
| 1. I get depressed when I think about how ageing might affect the things that I can do | _1_ | _2_ | _3_ | _4_ | _5_ |
| 1. The quality of my social life in later years depends on me | _1_ | _2_ | _3_ | _4_ | _5_ |
| 1. The quality of my relationships with others in later life depends on me | _1_ | _2_ | _3_ | _4_ | _5_ |
| 1. Whether I continue living life to the full depends on me | _1_ | _2_ | _3_ | _4_ | _5_ |
| 1. I get depressed when I think about the effect that getting older might have on my social life | _1_ | _2_ | _3_ | _4_ | _5_ |
| 1. As I get older there is much I can do to maintain my independence | _1_ | _2_ | _3_ | _4_ | _5_ |
| 1. Whether getting older has positive sides to it depends on me | _1_ | _2_ | _3_ | _4_ | _5_ |
| 1. Getting older restricts the things that I can do | _1_ | _2_ | _3_ | _4_ | _5_ |
| 1. Getting older makes me less independent | _1_ | _2_ | _3_ | _4_ | _5_ |
| 1. Getting older makes everything a lot harder for me | _1_ | _2_ | _3_ | _4_ | _5_ |
| 1. As I get older I can take part in fewer activities | _1_ | _2_ | _3_ | _4_ | _5_ |
| 1. As I get older I do not cope as well with problems that arise | _1_ | _2_ | _3_ | _4_ | _5_ |
| 1. Slowing down with age is not something I can control | _1_ | _2_ | _3_ | _4_ | _5_ |
| 1. How mobile I am in later life is not up to me | _1_ | _2_ | _3_ | _4_ | _5_ |
| 1. I have no control over whether I lose vitality or zest for life as I age | _1_ | _2_ | _3_ | _4_ | _5_ |
| 1. I have no control over the effects which getting older has on my social life | _1_ | _2_ | _3_ | _4_ | _5_ |
| 1. I get depressed when I think about getting older | _1_ | _2_ | _3_ | _4_ | _5_ |
| 1. I worry about the effects that getting older may have on my relationships with others | _1_ | _2_ | _3_ | _4_ | _5_ |
| 1. I go through cycles in which my experience of ageing gets better and worse | _1_ | _2_ | _3_ | _4_ | _5_ |
| 1. My awareness of getting older comes and goes in cycles | _1_ | _2_ | _3_ | _4_ | _5_ |
| 1. I feel angry when I think about getting older | _1_ | _2_ | _3_ | _4_ | _5_ |
| 1. I go through phases of feeling old | _1_ | _2_ | _3_ | _4_ | _5_ |
| 1. My awareness of getting older changes a great deal from day to day | _1_ | _2_ | _3_ | _4_ | _5_ |
| 1. I go through phases of viewing myself as being old | _1_ | _2_ | _3_ | _4_ | _5_ |

| **B) EXPERIENCE OF HEALTH-RELATED CHANGES** | | | | | |
| --- | --- | --- | --- | --- | --- |
| The next list describes some health-related changes you may have experienced. Can you tell me whether you have experienced these changes in the last 10 years and whether you believe that the changes experienced are specifically related to getting older or not. | | | | | |
|  |  | HAVE you experienced this change? | | ‘In terms of the changes you HAVE experienced: Do you think this change is ONLY related to, or due to the fact that, you are getting older’? | |
|  |  | Yes | No | Yes | No |
| Id1 | Weight problems | _1_ | _0_ | _1_ | _0_ |
| Id2 | Sleep problems | _1_ | _0_ | _1_ | _0_ |
| Id3 | Back problems or slipped disc | _1_ | _0_ | _1_ | _0_ |
| Id4 | Painful joints | _1_ | _0_ | _1_ | _0_ |
| Id5 | Not being mobile | _1_ | _0_ | _1_ | _0_ |
| Id6 | Loss of balance | _1_ | _0_ | _1_ | _0_ |
| Id7 | Loss of strength | _1_ | _0_ | _1_ | _0_ |
| Id8 | Slowing down | _1_ | _0_ | _1_ | _0_ |
| Id9 | Cramps | _1_ | _0_ | _1_ | _0_ |
| Id10 | Bone or joint conditions | _1_ | _0_ | _1_ | _0_ |
| Id11 | Cardiac or heart problems | _1_ | _0_ | _1_ | _0_ |
| Id12 | Ear or hearing problems | _1_ | _0_ | _1_ | _0_ |
| Id13 | Vision and eyesight changes | _1_ | _0_ | _1_ | _0_ |
| Id14 | Respiratory problems | _1_ | _0_ | _1_ | _0_ |
| Id15 | Foot problems | _1_ | _0_ | _1_ | _0_ |
| Id16 | Depression | _1_ | _0_ | _1_ | _0_ |
| Id17 | Anxiety | _1_ | _0_ | _1_ | _0_ |

***Please check that you have answered every question – thank you.***

©RCSI-2007 (email: psychology@rcsi.ie)
