# Supplementary material for: Cross-sectional validation of the Aging Perceptions Questionnaire: a multidimensional instrument for assessing self-perceptions of aging
Source: BMC Geriatr. 2007 Apr 26;7:9. doi: 10.1186/1471-2318-7-9 (PMC1868732; doi:10.1186/1471-2318-7-9)
Supplement: Additional file 2 — Scoring information. Protocol for tabulating APQ subscale scores. [file 1471-2318-7-9-S2.doc]

Additional scoring information

- - **The Ageing Perceptions Questionnaire (APQ)**
- **Views about getting older**
  - - Timeline acute/chronic: Mean of items 1, 2, 3, 4, and 5
    - Timeline cyclical: Mean of items 27, 28, 30, 31, 32
    - Emotional Representations: Mean of items 9, 13, 25, 26, 29
    - Control positive: Mean of items 10, 11, 12, 14, 15
    - Control negative: Mean of items 21, 22, 23, 24 (items are reverse-scored)
    - Consequences positive: Mean of items 6, 7, and 8
    - Consequences negative: Mean of items 16, 17, 18, 19, 20
- **Experience of health-related changes**
  - - Number of health-related changes experienced **=** sum of Id1a, Id2a, Id3a, Id4a, Id5a, Id6a, Id7a, Id8a, Id9a, Id10a, Id11a, Id12a, Id13a, Id14a, Id15a, Id16a, Id17a
    - Number of health-related changes attributed to ageing = sum of Id1b, Id2b, Id3b, Id4b, Id5b, Id6b, Id7b, Id8b, Id9b, Id10b,Id11b, Id12b, Id13b, Id14b, Id15b, Id16b, Id17b
    - Identity score: (number of changes attributed to ageing/ number of health-related changes experienced) x 100

Missing data protocol: On subscales with 5 items and 4 items we have allowed for a maximum of 2 missing items, i.e. if more than 2 items were missing then a subscale score was not calculated for that variable. For the 3 item subscale we have allowed for a maximum of 1 missing item.
